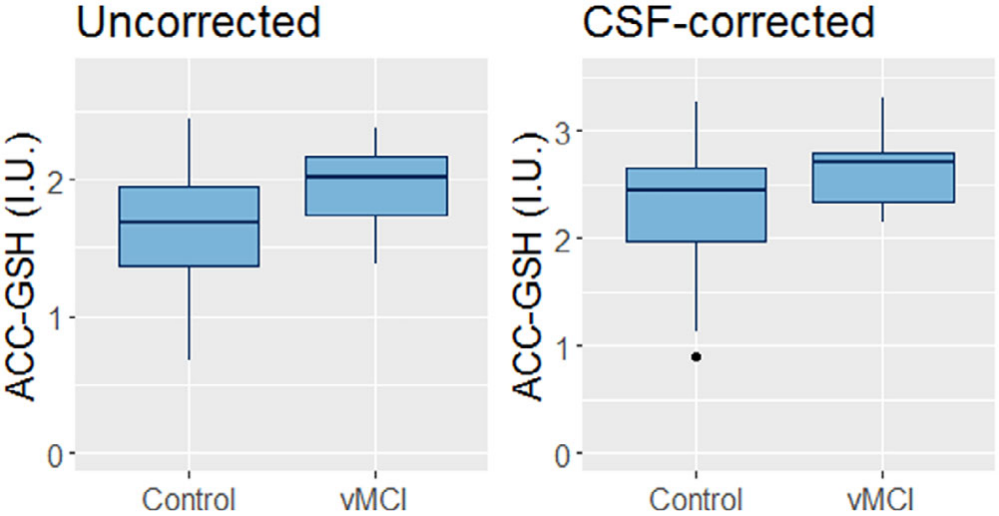# Brain glutathione in vascular mild cognitive impairment and cognitive correlates

**DOI:** 10.1002/alz.086397

**Published:** 2025-01-09

**Authors:** Jinghan Jenny Chen, Nathan Herrmann, Kate Survilla, Ethan Mah, Yejin Kang, Sandra E Black, Joel Ramirez, Ana C. Andreazza, Alex Kiss, Paul I. Oh, Damien Gallagher, Simon J. Graham, Krista L. Lanctôt

**Affiliations:** ^1^ University of Toronto, Toronto, ON Canada; ^2^ Neuropsychopharmacology Research Group, Sunnybrook Research Institute, Toronto, ON Canada; ^3^ Temerty Faculty of Medicine, University of Toronto, Toronto, ON Canada; ^4^ Hurvitz Brain Sciences Program, Sunnybrook Research Institute, Toronto, ON Canada; ^5^ Sunnybrook Research Institute, Toronto, ON Canada; ^6^ Division of Neurology, Department of Medicine, University of Toronto, Toronto, ON Canada; ^7^ LC Campbell Cognitive Neurology Research Unit, Sunnybrook Research Institute, University of Toronto, Toronto, ON Canada; ^8^ Sunnybrook Health Sciences Centre, Toronto, ON Canada; ^9^ Sandra Black Centre for Brain Resilience and Recovery, Sunnybrook Research Institute, Toronto, ON Canada; ^10^ Temerty Faculty of Medicine, Department of Psychiatry, University of Toronto, Toronto, ON Canada; ^11^ ICES, Toronto, ON Canada; ^12^ Department of Research Design and Biostatistics, Sunnybrook Research Institute, University of Toronto, Toronto, ON Canada; ^13^ KITE Research Institute, Toronto Rehabilitation Institute, Toronto, ON Canada; ^14^ Department of Medical Biophysics, University of Toronto, Toronto, ON Canada; ^15^ Department of Psychiatry, Temerty Faculty of Medicine, University of Toronto, Toronto, ON Canada; ^16^ Department of Pharmacology and Toxicology, University of Toronto, Toronto, ON Canada

## Abstract

**Background:**

Oxidative stress (OS) has been implicated in age‐related neurodegeneration and may be important in prodromal states such as vascular mild cognitive impairment (vMCI). Higher peripheral OS is reported in vMCI patients; however, the role of central antioxidant defenses in vMCI and their correlation to cognition is unclear. Glutathione (GSH) is a major brain antioxidant, and the current study assessed brain GSH in possible vMCI vs. controls.

**Methods:**

Possible vMCI patients (1 standard deviation (SD) below population norms in verbal memory, executive function (EF), processing speed, or working memory, age 55‐85, and currently enrolled in a 6‐month exercise rehabilitation program due to having 2 or more vascular risk factors or previous vascular event) and cognitively‐normal (CN) controls were recruited. All participants received 1H magnetic resonance spectroscopy (MEscher–GArwood Point Resolved Spectroscopy) to quantify brain GSH at baseline in the anterior cingulate (AC) and occipital lobe (OL). Spectroscopic analysis was completed using the Gannet toolkit (vers. 3.1) in Matlab (vers. 2020b).

**Results:**

In 43 participants (mVCI n = 22, CN n = 21), AC‐GSH (I.U. ± SD) was higher in mVCI (1.96 ± 0.29) compared to CN (1.64 ± 0.48) (F_(1,29.7)_ = 6.9, p = .01); this difference remained after correcting for cerebrospinal fluid (CSF) volume (F_(1,28.4)_ = 6.5, p = .02), and controlling for age and sex (B [SE] = 0.33 [0.11], p = .007). There was no difference in OL‐GSH before or after correcting for CSF volume. Higher AC‐GSH, but not OL‐GSH, was correlated with poorer global cognition (Montreal Cognitive Assessment ‐Full score, B [SE] = 2.15, p = .03), and poorer EF performance (B [SE] = ‐0.67 [0.25], p < .001). These relationships remained significant after correcting for CSF volume.

**Conclusion:**

The current study suggests an upregulation of AC glutathione in vMCI, which may reflect a compensatory increase in antioxidants as a response to oxidative stress challenges reported in these patients. Higher brain GSH in the AC region is correlated with poorer global cognition and executive function performance, suggesting a link between local brain antioxidant response and disease‐relevant cognitive domains.